# Adolescents' health and health behaviour as predictors of injury death. A prospective cohort follow-up of 652,530 person-years

**DOI:** 10.1186/1471-2458-8-90

**Published:** 2008-03-17

**Authors:** Ville M Mattila, Jari Parkkari, Leena Koivusilta, Tapio Nummi, Pekka Kannus, Arja Rimpelä

**Affiliations:** 1 School of Public Health, University of Tampere, 33014 Tampere, Finland; 2 Department of Orthopaedic Surgery, University Hospital of Tampere, 33014 Tampere, Finland; 3 Tampere Research Centre of Sports Medicine, UKK Institute, 33501 Tampere, Finland; 4 University of Tampere, University of Turku, Finland, IASM – Institutions and Social Mechanisms Faculty of Social Sciences, 20014 University of Turku, Finland; 5 Injury & Osteoporosis Research Centre, UKK Institute, 33014 Tampere, Finland

## Abstract

**Background:**

Injuries represent an important cause of mortality among young adults. Longitudinal studies on risk factors are scarce. We studied associations between adolescents' perceived health and health behaviour and injury death.

**Methods:**

A prospective cohort of 57,407 Finns aged 14 to 18 years was followed for an average of 11.4 years. The end-point of study was injury death or termination of follow-up in 2001. The relationships of eight health and health behaviour characteristics with injury death were studied with adjusted Cox's proportional hazard model.

**Results:**

We identified 298 (0.5%) injury deaths, 232 (0.9%) in men and 66 (0.2%) in women. The mean age at death was 23.8 years. In the models adjusted for age, sex and socioeconomic background, the strongest risk factors for injury death were recurring drunkenness (HR 2.1; 95% CI: 1.4–3.1) and daily smoking (HR 1.7; 95% CI: 1.3–2.2). Poor health did not predict injury death. Unintentional and intentional injury deaths had similar health and health behavioural risk factors.

**Conclusion:**

Health compromising behaviour adopted at adolescence has a clear impact on the risk of injury death in adulthood independent from socioeconomic background. On the other hand, poor health as such is not a significant predictor of injury death. Promotion of healthy lifestyle among adolescents as part of public health programmes would seem an appropriate way to contribute to adolescent injury prevention.

## Background

Injuries are an important cause of morbidity and mortality among the young worldwide [1-4]. Previous studies provide evidence of an association between injury-related deaths among young people and lower socioeconomic status [5-7], low education of household [5] and living in rural areas [8]. Concern has also been directed to the association between health and health behaviours and injury death. It seems evident that use of alcohol increases the risk of injury death [9,10]. In addition, emotional instability [11] has been associated with an increased injury risk. In cross-sectional investigations, risk taking behaviour, physical activity and team sports were also related to injuries [12,13]. Using cross-sectional study designs, the authors have previously shown that poor health and health compromising behaviour are significant risk factors for less severe injuries, while socioeconomic status is only weakly related to injuries in Finland [14,15].

Health behaviours might be expected to have a two-way relationship with injury risk; a direct causal effect on injury risk (such as drunkenness) or they can be indicators of health compromising and risk taking behaviour (such as smoking) [13,16]. Smoking and drinking have previously been associated with injuries [10,17-19]. However, it is not known whether this association reflects underlying differences in the socioeconomic status between the groups, or whether they have an independent predictive effect on injury risk. High intensity sport, on the other hand, has been shown to be associated with an increased risk of injury [20], probably resulting from the increased intensity of physical activity and longer exposure times. Alternatively, its effect can be explained by selection, i.e. the inclination of competitive, risk-taking persons to attend sports clubs.

From the health point of view, adolescence is an important stage of life. Several health compromising behaviours (e.g. smoking, alcohol) as well as health enhancing behaviours (e.g. physical exercise) are adopted in adolescence and they often persist into adulthood [21]. Furthermore, compulsory schooling ends at late adolescence. In previous studies, poor school success has been associated with various health-compromising behaviours including violence [22] and problem drinking [23]. It is not known whether these factors predict injuries in later life or are merely reflections of socioeconomic differences causing the variation in injury risk. We hypothesised that health compromising (such as drinking and smoking) and competitive, risk-taking (such as attending sport clubs) behaviours are independent risk factors for injury death in young adulthood.

By combining population surveys to a cause-of-death register in a longitudinal study design our first aim was to investigate whether adolescents' health and health behaviour predict injury death later in life and whether this association is dependent on socioeconomic background and school success. Our second aim was to compare the risk factors for unintentional and intentional injury deaths in this age group.

## Methods

### Baseline cohort

Using the opportunity to combine questionnaire survey data with data from a national register, we constructed a longitudinal study design to obtain information on the predictive factors for injury deaths among adolescents. The biennial Adolescent Health and Lifestyle Survey (Finland) is a nationwide monitoring system of health and health-related lifestyle conducted by mailed questionnaires since 1977. Two re-inquiries are sent to non-respondents after three and seven weeks. The materials with respect to sampling, research methods, questions, and time of inquiry were maintained as similar as possible for each year. The questionnaire is available at internet [24]. The sample of 14, 16 and 18-year-olds is drawn from the National Population Register Centre through selection of all Finns born on certain days in June, July or August. The mean ages of the respondents are 14.6, 16.6 and 18.6 years. Baseline data for our analyses were collected from 1979 to 1997. The baseline cohort consisted of 72,378 persons, of whom 57,407 responded to the survey, the response rate being 79% (Table 1).

**Table 1:** Age, number, and response rates of 72,378 Finns in 1979–2001.

**Age at follow-up in 2001 (years)**	**Age at baseline (years)**	**Baseline year**	**Number of participants**		**Response rate (%)**	
			
			Boys	Girls	Boys	Girls
37	14	1979	564	535	86	91
39	16		528	577	83	91
41	18		528	512	78	86
35	14	1981	488	548	87	92
37	16		535	529	85	92
39	18		518	524	81	88
33	14	1983	429	482	79	86
35	16		414	511	75	91
	18		-*	-*	-	-
31	14	1985	395	433	75	88
33	16		455	499	77	87
35	18		408	470	67	83
29	14	1987	1,674	1,789	81	89
31	16		1,383	1,479	80	89
33	18		1,012	1,274	74	89
27	14	1989	360	431	75	90
29	16		362	380	70	82
31	18		326	407	63	80
25	14	1991	1,629	1,837	74	87
27	16		1,562	1,912	71	87
29	18		1,286	1,626	61	82
23	14	1993	1,861	2,008	75	88
25	16		1,655	1,943	71	87
27	18		1,460	1,791	67	84
21	14	1995	1,177	1,301	75	85
23	16		1,232	1,469	72	88
25	18		1,071	1,313	67	86
19	14	1997	1,168	1,346	69	84
21	16		1,126	1,379	68	87
23	18		1,088	1,414	60	83

		Total	26688	30719	72	87

* In 1983, no persons aged 18 were included in the sample

### Follow-up injury data

The starting point of follow-up was the day of the survey end point, April 30 of each survey year. For our study, the end points were injury death, other death, or termination of the study on December 31, 2001. The respondents were followed for an average of 11.4 years, with the total follow-up time of 652,530 person-years. Since the follow-up time ranged from 0 to 23 years, the participants' age ranged from 14 to 41 years at the end of follow-up (Table 1). Persons deceased for reasons other than injury (N = 123) during the study period were followed up to the time of death.

Injury-related death data were obtained from the Official Cause-of-Death Statistics, which is a statutory, computer-based register covering the entire population of the country [25]. The main categories for unintentional injury death in this register are road traffic accidents, water traffic accidents, falls, drownings and poisonings. Suicides and homicides are the main categories of death for intentional injuries [25]. We linked our baseline cohort with this register by means of the unique national personal identification numbers. Approval was obtained from the Institutional Review Board of the Statistics Finland (TK-53-1526-04).

The Finnish Official Cause-of-Death Statistics are in practice 100% complete, since each death, its certificate and the corresponding personal information in our computerised population database are cross-checked. Furthermore, the accuracy of the data is maximised by a 3-phase process, in which each death certificate and its codes are cross-examined [25,26]. In injury-based deaths, the accuracy of the Finnish death certificates and their cause-of-death codes are verified further by autopsies performed on 94% to 97% of these deaths [25,26].

### Health and health behaviour indicators at baseline

In this study, we explored the relationships of eight health and health behavioural categorical variables with injury death.

For the respondents' perception of their own health in general three categories were used: excellent (31%), good (49%), and average or poor (20%). Chronic disease or disability restricting daily activities was asked with "no" (89%) or "yes" (11%). A summary index of weekly perceived stress symptoms (stomach ache, tension, irritability, sleep difficulty, headache, trembling of hands, feeling tired or weak, feeling dizzy) were calculated as: none (41%), one (21%), two (15%), and three or more (23%). Body mass index (BMI) was calculated by dividing weight (kg) with the square of height (m). The cut-off points to describe overweight (no 89%, yes 11%) were set according to Cole and colleagues [27].

Adolescents' health behaviours were described by daily use of tobacco (no 77%, yes 23%) and drinking style (abstinence 23%, occasional drinking [once a month but not until drunkenness] 30%, recurrent drinking [more than once a month but rarely until drunkenness] 30%, and recurring drunkenness [weekly] 17%). Frequency of participation in organised sports and other leisure time physical activities was asked with following categories: never (59% and 5%), 2 to 3 times a week or less (30% and 63%) and 4 or more times a week (11% and 32%, respectively).

### Covariates

Sociodemographic background was adjusted using three variables; father's or other guardian's education (high 17%, middle 16%, low 70%), respondent's family structure (both parents 77%, other 23%), and urbanisation level of residence (capital area 11%, large town 8%, small town 35%, village 31%, sparsely populated rural municipality 16%).

Respondents' school success at age 14 was measured by the pupil's own assessment of his or her position in class according to the average grade in the preceding end-of-term school report (good, better than average, lower than average, poor). For the age groups 16 and 18, school success was determined as the combination of the school type attended (upper secondary, vocational school, trade course, course for unemployed) and school success (excellent, good, satisfactory, poor). Respondents who attended upper secondary school and reported excellent or good school success were categorised as having excellent school success. Good school success category included other adolescents from upper secondary school and adolescents from vocational school whose school success was excellent or good. Adolescents with poorer grades in vocational school were categorised as having satisfactory school success. Poor school success category included adolescents who attended trade course or course for unemployed.

### Statistical methods

Cox's proportional hazard models were used to analyse associations between baseline characteristics and unintentional injury death, intentional injury death and alcohol-related injury death. The modelling approach was based on existing literature (see Introduction) and on our hypothesis that health and health behaviours measured in adolescence are associated with unintentional and intentional injury death later in life independently from socioeconomic status. First, the hazard ratios of injury deaths were adjusted for age at baseline and stratified by sex, since the hazard ratio was not constant between sexes. Since the predictors of unintentional versus intentional injury death were not markedly different, all injury deaths were also analysed combined. Second, father's or other guardian's education, respondent's family structure and urbanisation level of residence were added to the model (previous literature has shown the association between socioeconomic status, urbanisation level of residence and injury death). The final analysis included all the aforementioned variables and school success (which has shown to be associated with persons' health in general). In all models, hazard ratios (HR) were calculated with 95% confidence intervals (95% CI).

Considering the long follow-up of 20 years, the baseline data were divided into three time periods (1979–1985, 1986–1991, 1992–1997) and for each time period, separate Cox regression models were calculated. Since no difference over time was seen (data not shown), all data were combined for the above noted analyses.

## Results

In the initial analysis for the baseline cohort, we identified 298 (0.5%) injury-related deaths. Men (232, 0.9%) were significantly more likely to experience injury death than women (66, 0.2%) (p < 0.001). Of the deaths among men, 109 (47%) were unintentional and 123 (53%) intentional, while the corresponding figures among women were 27 (41%) and 39 (59%). Suicides were the leading cause-of-death in intentional injury deaths (117 men, 96.7%; 36 women, 92.3%). Table 2 shows the distribution of injury categories. One-third of injury deaths (101/298) were alcohol-related, as judged from the autopsy reports. The mean age at the time of injury death was 23.8 years.

**Table 2:** Injury deaths by injury categories and alcohol use among 57,407 respondents during follow-up.

**Injury category**	**Number of alcohol-related deaths (%)**	**Total number (%)**
Road traffic accidents	23 (23%)	87 (28%)
Water traffic accidents	3 (3%)	5 (2%)
Falls	3 (3%)	5 (2%)
Drownings	3 (3%)	8 (3%)
Poisonings	1 (1%)	17 (5%)
Other unintentional injury	6 (6%)	14 (5%)
**Unintentional deaths, total**	**39 (39%)**	**136 (44%)**
Suicides	58 (58%)	153 (49%)
Homicides	4 (4%)	9 (3%)
**Intentional deaths, total**	**62 (61%)**	**162 (52%)**

**Total**	**101 (100%)**	**298 (100%)**

Non-respondents at baseline had more injury-related deaths (n = 146) in the follow-up than respondents (n = 298) (1.0% vs. 0.5%) (p < 0.001). Of the 146 deaths among non-respondents, 72 (49.3%) were unintentional and 74 (50.7%) intentional. The distribution by injury type was similar to that among respondents (Table 2). Taking gender and response into account, our analysis showed that 1.4% of non-respondent men died of injurious causes compared with 0.9% of respondent men (p = 0.005). The corresponding proportions for women were 0.2% and 0.2%, respectively.

Age-adjusted, sex-stratified hazard ratios for unintentional injury death and intentional injury death are shown in Table 3. The strongest predictor of both unintentional and intentional injury deaths was recurring drunkenness (HR 2.6; 95% CI: 1.6–4.5 for unintentional injury death and HR 2.7; 95% CI: 1.6–4.5 for intentional injury death). Smoking predicted significantly both injury deaths. Of the four health variables (perceived health, overweight, stress symptoms and chronic disease) only the number of stress symptoms weekly (HR 1.7; 95% CI 1.1–2.6) and poor self-perceived health (HR 1.8, 95% CI: 1.3–3.0) were significantly associated with intentional injury deaths. None of the health variables were significantly associated with unintentional injury deaths. Due to the similarities among the risk factors, unintentional and intentional injury deaths were combined in further analysis.

**Table 3:** Hazard ratios for unintentional and intentional injury deaths during follow-up by health and health behaviour variables at baseline. Results are adjusted for age and stratified by sex.

**Background variable at the baseline**	**Unintentional injury death (N = 136)**	**Intentional injury death (N = 162)**
Perceived health status		
Excellent	1	1
Good	0.9 (0.6–1.5)	1.3 (0.8–1.9)
Poor	1.2 (0.7–2.0)	**1.8 (1.2–3.0)**
Chronic disease or disability		
No	1	1
Yes	1.0 (0.6–1.9)	1.3 (0.8–2.1)
Number of stress symptoms weekly		
0	1	1
1	1.0 (0.6–1.6)	1.0 (0.6–1.6)
2	1.3 (0.8–2.3)	1.6 (1.0–2.7)
3+	1.3 (0.8–2.2)	**1.7 (1.1–2.6)**
Overweight		
No	1	1
Yes	1.3 (0.8–2.0)	0.8 (0.5–1.4)
Smoking		
Not daily	1	1
Daily	**1.9 (1.4–2.7)**	**2.3 (1.7–3.2)**
Drinking style		
Abstinence	1	1
Occasional drinking	1.2 (0.8–2.1)	1.3 (0.8–2.2)
Recurrent drinking	1.7 (1.0–2.9)	1.5 (0.9–2.5)
Recurring drunkenness	**2.6 (1.6–4.5)**	**2.7 (1.6–4.5)**
Frequency of participation in organised sports		
Never	1	1
2–3 times a week or less	1.0 (0.7–1.7)	0.7 (0.5–1.0)
4–5 times a week or more	0.8 (0.4–1.4)	**0.5 (0.2–0.9)**
Frequency of other leisure-time physical exercise		
Never	1	1
2–3 times a week or less	0.4 (0.2–1.1)	0.7 (0.3–1.8)
4–5 times a week or more	0.4 (0.2–1.0)	0.7 (0.3–1.7)

When socioeconomic background and school success were taken into account. frequent drunkenness as drinking style lost its significance slightly as a predictor of injury death. Nonetheless, it remained the strongest health behavioural predictor of injury death (HR 2.1; 95% CI: 1.4–3.1) (Table 4). Smoking was another significant predictor of injury death when socioeconomic status and school success were taken into account (HR 1.7; 95% CI: 1.3–2.2). Frequent participation in other (than sport club) leisure-time physical exercise seemed to decrease the risk of injury death (HR 0.5, 95% CI: 0.3–0.9) (Table 4), but its effect was lost after adjusting for socioeconomic background. Health status was not a significant predictor of injury death in multivariate models.

**Table 4:** Hazard ratios for injury death by health and health behaviour variables at baseline. Results are adjusted for age and socioeconomic background** and stratified by sex.

**Background variable**	**All injury deaths (N = 298)**		
	*	**	***
Perceived health status			
Excellent	1	1	1
Good	1.1 (0.8–1.4)	1.0 (0.8–1.4)	0.9 (0.7–1.3)
Poor	**1.5 (1.1–2.1)**	1.3 (0.9–1.9)	1.2 (0.9–1.7)
Chronic disease or disability			
No	1	1	1
Yes	1.2 (0.8–1.7)	1.0 (0.7–1.5)	1.0 (0.7–1.5)
Number of stress symptoms weekly			
0	1	1	1
1	1.0 (0.7–1.4)	1.0 (0.7–1.4)	1.0 (0.7–1.5)
2	**1.5 (1.1–2.1)**	1.3 (0.9–1.9)	1.4 (0.9–2.0)
3+	**1.5 (1.1–2.0)**	1.4 (1.0–1.9)	1.4 (1.0–2.0)
Overweight			
No	1	1	1
Yes	1.0 (0.7–1.5)	1.0 (0.7–1.5)	1.0 (0.7–1.5)
Smoking			
Not daily	1	1	1
Daily	**2.1 (1.6–2.6)**	**1.9 (1.5–2.5)**	**1.7 (1.3–2.2)**
Drinking style			
Abstinence	1	1	1
Occasional drinking	1.3 (0.9–1.8)	1.2 (0.8–1.7)	1.1 (0.8–1.0)
Recurrent drinking	**1.6 (1.1–2.2)**	**1.5 (1.1–2.2)**	1.5 (1.0–2.1)
Recurring drunkenness	**2.6 (1.8–3.8)**	**2.1 (1.4–3.4)**	**2.1 (1.4–3.1)**
Frequency of participation in organised sports			
Never	1	1	1
2–3 times a week or less	0.8 (0.7–1.1)	0.8 (0.6–1.1)	0.9 (0.7–1.1)
4–5 times a week or more	**0.6 (0.4–0.9)**	0.7 (0.4–1.1)	0.7 (0.4–1.2)
Frequency of other leisure-time physical exercise			
Never	1	1	1
2–3 times a week or less	**0.5 (0.3–0.9)**	0.6 (0.3–1.1)	0.7 (0.4–1.3)
4–5 times a week or more	**0.5 (0.3–0.9)**	0.6 (0.3–1.1)	0.7 (0.4–1.4)

*Adjusted for age, and stratified by sex

** Adjusted for age, sex, father's or other guardian's education, respondent's family structure, and urbanisation level of residence

*** Adjusted for aforementioned and school success

When alcohol-related injury deaths (n = 101) were analysed separately, the strongest risk factor was the drinking style. Those reporting recurring drunkenness had 4.2 (95% CI: 2.1–8.5) times the risk of injury death. The increased risk remained significant after adjusting the analysis for age, sex, father's or other guardians' education, respondent's family structure, urbanisation level of residence and school success (HR 3.1; 95% CI: 1.5–6.4). Smoking was also a significant risk factor for alcohol-related injury death (adjusted HR 2.1; 95% CI: 1.2–3.9)

## Discussion

This prospective adolescent cohort study, to our knowledge the largest and longest injury follow-up ever documented, showed that health compromising behaviours in adolescence predict injury death during transition to adulthood. The predictive strength of health behavioural risk factors remained significant after adjusting for socioeconomic status, while poorer health lost its predictive strength after adjusting for socioeconomic background. High-intensity physical activity in sports clubs was not associated with the risk of injury death. Risk factors for unintentional and intentional injury deaths did not differ significantly. Thus, health compromising behaviour in adolescence seems to reflect an increased risk of injury death in adulthood, independently from socioeconomic status and irrespective of injury type.

This study had several strengths. Firstly, it involved a unique, prospective, nationwide sample of adolescents over a substantial follow-up period of 652,530 person-years. Secondly, the Finnish Official Cause-of-Death Statistics is a very complete and accurate register for epidemiologic purposes [25,26]. Thirdly, the used background variables have shown good repeatability [28].

There were also some limitations. Although the overall response rates were good, they somewhat declined over the years, from 85% in 1979 to 75% in 1997. The analysis of non-respondents showed that especially non-responding boys had more injury deaths than those who responded. This could be explained by a higher risk profile related to the association between non-response and health-compromising behaviour [29]. The higher injury rate among non-respondents may have resulted in underestimation of the predictive strength of the examined risk factors. On the other hand, the distribution of injury type did not differ between respondents and non-respondents.

In this longitudinal study, health compromising behaviours (smoking and drinking) were associated with an increased risk of injury death – as has previously been suggested by results from cross-sectional studies [17-19,30]. Our finding thus confirmed that alcohol use increases the risk for injury death [10,31]. This finding is particularly important in Finland, where drunkenness figures among adolescents have been rated the third highest of 30 European countries [32].

Adolescent smoking and drinking are behaviours that easily persist into adulthood [21]. Consequently, although we did not have information about respondents' adult drinking and smoking habits, it was not surprising that the strength of drinking and smoking as predictors of injury death likewise persisted beyond adolescence. Furthermore, it is likely that these risky persons are characterised by additional health compromising and risk-taking behaviours, which may also contribute to the increased injury risk. On the other hand, since the association between alcohol, smoking and injuries remained significant even when socioeconomic status and school success were adjusted for, we feel that adolescent drinking and smoking, as such, should be considered as serious indicators of risk-taking behaviour and subsequent injuries.

From the point of view of injury prevention, any major conclusion concerning causality between health compromising behaviour and injuries should be drawn with caution. Although our results are fairly convincing in showing that health compromising behaviour adopted at adolescence has a clear impact on the risk of injurious death in early adulthood, no preventive programme should be launched based on our findings alone. Intervention literature provides some evidence that brief alcohol intervention among injured persons with alcohol abuse may reduce injury occurrence [33].

Contrary to our hypothesis, participation in organised sports was unrelated to injury death, even when unintentional injury deaths were independently analysed. The existing evidence shows that sports injuries account for a significant proportion of adolescent non-fatal injuries [20,34]. It is obvious that persons participating in organised sports are more competitive. However, this competitiveness does not seem to increase the person's injury death risk.

Cross-sectional studies have provided some evidence that poor perceived health is associated with injuries [14]. Our current study showed that reporting several stress symptoms in adolescence was associated with future injury-related death, but the significance was lost when socioeconomic background was taken into account. In addition, intentional injury death showed a weak association with poorer than average health. However, the risk factors for unintentional and intentional injury deaths (mainly suicides) did not differ markedly. It is possible that the baseline survey response rates were lower for persons with severe mental health problems leading to later suicide, thus resulting in selection bias. Further research is therefore warranted to shed more light on the association between poor health and injury risk.

## Conclusion

In conclusion, health compromising behaviour adopted at adolescence has a clear impact on the risk of injury death in adulthood and this relationship is independent from the socioeconomic background. On the other hand, poor health as such is not a significant predictor of injury death. Promotion of healthy lifestyle among adolescents as part of public health programmes would seem an appropriate way to contribute to adolescent injury prevention, with a potential to carry the good effect into adulthood as well.

## Competing interests

The author(s) declare that they have no competing interests.

## Authors' contributions

AR, LK, JP and VM designed and set up the cohort and designed this study. VM performed the analysis and wrote the first draft of the report. TN and PK helped design the trial and contributed to the statistical analyses of this paper. All authors contributed equally to the drafting and editing of the study and assisted in finalising the report.

## Pre-publication history

The pre-publication history for this paper can be accessed here:


